# Uniqueness of radiomic features in non‐small cell lung cancer

**DOI:** 10.1002/acm2.13787

**Published:** 2022-09-29

**Authors:** Gary Ge, Jie Zhang

**Affiliations:** ^1^ Department of Radiology University of Kentucky Lexington Kentucky 40536 USA

**Keywords:** concordance correlation coefficient, feature uniqueness, lung cancer, NSCLC, radiomic features

## Abstract

**Purpose:**

The uniqueness of radiomic features, combined with their reproducibility, determines the reliability of radiomic studies. This study is to test the hypothesis that radiomic features extracted from a defined region of interest (ROI) are unique to the underlying structure (e.g., tumor).

**Approach:**

Two cohorts of non‐small cell lung cancer (NSCLC) patients were retrospectively retrieved from a GE and a Siemens CT scanner. The lung nodules (ROI) were delineated manually and radiomic features were extracted using IBEX. The same ROI was then translocated randomly to four other tissue regions of the same set of images: adipose, heart, lung beyond nodule, and muscle for radiomic feature extraction. Coefficient of variation (CV) within different ROIs and concordance correlation coefficient (CCC) between lung nodule and a given tissue region were calculated to test to determine feature uniqueness. The radiomic features were considered nonunique when (1) the CV < 10% and CCC > 0.85 for over 50% of patients; and (2) the CCC > 0.85 appeared in ≥2 tissue regions beyond the defined region.

**Results:**

In total, 14 patients from GE and 18 patients from Siemens are analyzed. The results show that 12 features fall below the 10% CV threshold for over 50% of patients in the GE cohort and 29 features in the Siemens cohort. According to CCC, 18 radiomic features in GE and 16 features in Siemens are identified as nonunique, with 11 overlapping features. Combining CV and CCC, 9 of 123 calculated features (7.3%) are identified as nonunique to a defined ROI.

**Conclusions:**

Radiomic feature uniqueness should be considered to improve the reliability of radiomics study.

## 1 INTRODUCTION

Radiomics is a method that extracts a large amount of features from radiographic medical images using data‐characterization algorithms.^1^ It is a rapidly advancing field of clinical image analysis with a vast potential for supporting decision‐making involved in the diagnosis and treatment of cancer.^2^, ^3^, ^4^, ^5^, ^6^, ^7^, ^8^, ^9^, ^10^, ^11^, ^12^, ^13^, ^14^, ^15^, ^16^, ^17^

In radiomics, the extracted image features, termed radiomic features, are usually generated from a segmented volume (2D or 3D). The mathematically extracted high‐dimensional feature data (semantic features such as shape and spiculation and agnostic features such as kurtosis and texture) are used to quantitatively describe attributes of a region of interest (ROI), which is a tumor in most cases.^1^, ^18^ Since its introduction, radiomic studies have experienced an exponential increase in popularity. However, its clinical applications are far behind. One of the major challenges that hamper clinical applications of radiomic features is their reproducibility. This challenge has been recognized recently and more studies have dedicated to investigate the impact of various factors (e.g., acquisition parameters and reconstruction algorithms) on the reproducibility of radiomic features.^19^, ^20^, ^21^, ^22^

Besides feature reproducibility, feature uniqueness may be of another concern that could directly impact radiomic studies. Feature uniqueness can be defined as radiomic features that are solely attributed to the ROI from which it is extracted. Radiomic feature values are calculated based on pixel values inside a defined ROI. It is expected that feature values extracted from different locations within an image set will be different, even with the ROI kept the same. When a feature value extracted from an ROI is distinguishable from those extracted from other locations within the same image set, the feature can be considered to be unique to the ROI. When feature values from different locations, including the ROI, are comparable, the feature is not unique to the ROI. Radiomic features that are not unique to the target ROI should be excluded from radiomic studies. This is because the nonunique radiomic features do not provide information that is specific to the ROI and instead may introduce irrelevant information.

Feature uniqueness is different from feature reproducibility, which tackles the concern if a feature is reproducible when acquisition parameters or subjects change within a given *study cohort*.^20^, ^21^, ^22^ A unique feature is not necessarily reproducible, and a reproducible feature is not necessarily unique either. As radiomic features are extracted from the segmented volume (typically a tumor), they are naturally assumed to be unique to the underlying structure. However, to our knowledge, this assumption is not yet verified.

This study is proposed to test the hypothesis that radiomic features extracted from a defined ROI solely belong to the underlying structure (e.g., tumor). We use CT images of non‐small cell lung cancer (NSCLC) as an example. The presented method can be readily applied to other types of cancer and different imaging modalities for future studies.

## 2 METHODS

### 2.1 Study design

To test the hypothesis if radiomic features are unique to the region (in this study, lung nodule) where they were extracted, we performed a retrospective study of patients with NSCLC through comparisons of radiomic features in the lung nodule and other regions within the same image or image set. The rationale was that if the radiomic features of the lung nodule were unique, image features in other regions beyond the nodule would be different. Statistically speaking, there should be no correlation between radiomic features in the nodule and those in other regions. To enhance the robustness of the test, we chose four non‐tumor ROIs for statistical analysis. The same delineated ROI was used for all the regions to eliminate potential effects of shape on feature values.

**FIGURE 1:**
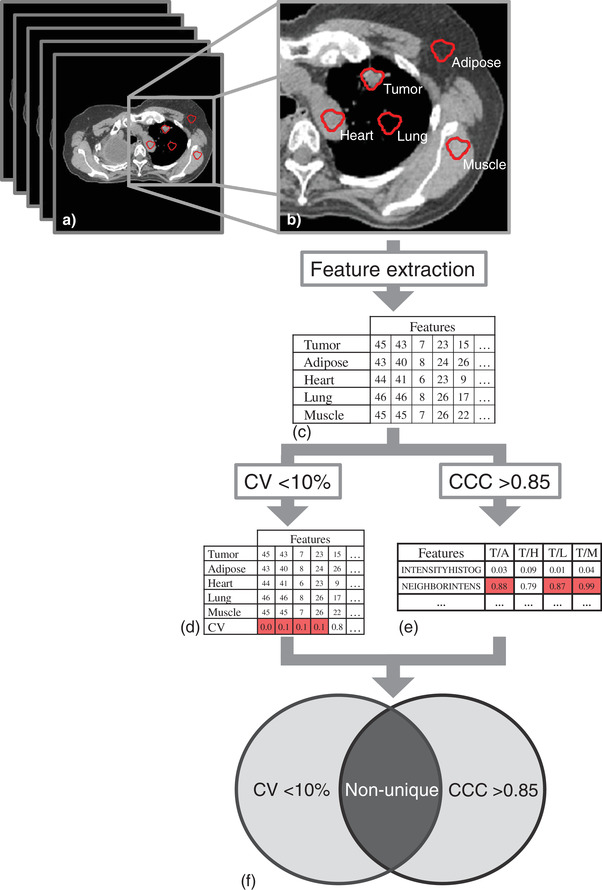
Illustration of the study flow: (a) CT images are retrieved and the lung nodule is segmented as the region of interest (ROI); (b) the same ROI was translocated to other regions: lung beyond tumor, adipose, heart, and muscle, respectively; (c) radiomic features were extracted from five ROIs; (d) statistical analysis. Coefficient of variation (CV) is calculated for each feature using the feature values for all five tissues, and (e) pairwise concordance correlation coefficient (CCC) is calculated for all four tumor/non‐tumor pairs (tumor/adipose, tumor/heart, tumor/lung, and tumor/muscle); (f) CV and CCC are used to determine feature uniqueness.

Figure 1 illustrates the study workflow. First, the lung nodule was segmented and the ROI was defined. Second, the same ROI was translocated to other regions: lung beyond tumor, adipose, heart, and muscle. Third, radiomic features were extracted from the five ROIs. Last, the correlation and variation between features in the nodule and other regions were calculated to identify if there were correlations between those image features in different regions. This retrospective study was approved by the Institutional Review Boards of the University of Kentucky.

### 2.2 Patient population and image acquisition

CT images were retrospectively retrieved from patients with NSCLC who underwent CT examinations in two different CT scanners, a GE VCT (GE Healthcare, Chicago, IL) and a Siemens SOMATOM Force (Siemens Healthineers, Erlangen, Germany).

For the GE VCT, CT images were retrieved from patients who were previously diagnosed with lung cancer and were scanned for radiation therapy treatment planning. The acquisition protocol was with 120 kV, collimation of 16 × 0.625 mm, and scan FOV of 50 cm with AutomA/SmartmA “on.” Filtered BackProjection and Standard kernel were used to reconstruct the images with a slice thickness of 2.5 mm. For the Siemens Force, CT images were retrieved from patients who were scanned with a low‐dose lung cancer screening protocol. The acquisition parameters were 100 kV, collimation of 192 × 0.6 mm, scan FOV of 50 cm with CAREDose “on.” The iterative reconstruction (ADMIRE) and BR40 kernel were used to reconstruct CT images with a slice thickness of 0.5 mm and reconstruction FOV of 35 cm.

### 2.3 Image segmentation and ROI translocation

ROIs were manually delineated over the primary tumor for all image sets and represented 3D segmentations. All segmentations were performed using Eclipse (Varian, Palo Alto, USA) for the GE VCT and IBEX (MD Anderson, Houston, USA) for the Siemens Force.^23^ The segmentations on images acquired with the GE VCT were direct from radiation therapy treatment planning contours. Segmentations on images acquired with the Siemens Force were performed by the researchers. In this study, feature uniqueness was evaluated using the same ROI as the target lung nodule. Variation in segmentation was not considered as there was no potential impact on the feature comparison.

After the tumor ROI (ROI_T_) was identified, the ROI_T_ (*x, y*) coordinates were translocated in‐plane to generate four additional ROIs, each located within a different tissue region. The four tissue types chosen were adipose, heart, lung, and muscle. The corresponding ROIs were named ROI_A_, ROI_H_, ROI_L_, or ROI_M_, respectively. In circumstances where there were no viable locations in‐plane to generate one of the additional ROIs, an appropriate *z*‐location was chosen before repeating the translocation process to acquire the remaining non‐tumor ROIs. The ROI translation was completed using a self‐developed program on MATLAB 2020a (MathWorks, Natick, USA).

### 2.4 Feature extraction

Radiomic features were extracted from each ROI with IBEX.^23^ Our goal was to compare radiomic features extracted from different ROIs within the same image set. No image preprocessing was done prior to feature extraction. Any preprocessing would have had no influence on comparison, as the potential impact of preprocessing on the image would be simultaneously applied to all ROIs within the image set. A total of 123 features were obtained using IBEX. Some features were directionally dependent or percentile‐based and were calculated separately for each direction or percentile, respectively. When counting all versions of the extracted features, a total of 1739 feature values were obtained.

The extracted features were categorized as Intensity Direct features (*n* = 34), Intensity Histogram features (*n* = 9), Intensity Histogram Gauss Fit features (*n* = 6), 2D and 3D Gray Level Cooccurrence features (*n* = 44), Gray Level Run Length features (*n* = 11), 2D and 3D Neighbor Intensity Difference features (*n* = 10), and Gradient Orient Histogram features (*n* = 9). Shape features were also generated using IBEX, but these were excluded from analysis as the shape features were intrinsically unique to a given ROI.

### 2.5 Statistical analysis

To identify nonunique features, coefficient of variation (CV) and concordance correlation coefficient (CCC) were calculated for comparison analysis. For each image set, CV was calculated for each radiomic feature using values from all defined ROIs. A CV of <10% was chosen as the threshold to indicate feature reproducibility.^24^, ^25^, ^26^, ^27^

CCCs were calculated between the tumor and each of four other regions, in total four pairs: tumor–adipose (T–A), tumor–heart (T–H), tumor–lung (T–L), and tumor–muscle (T–M). For each pair, CCC was calculated for each feature category (e.g., Intensity Direct and Gradient Orient Histogram). For features that had directional‐dependence or percentile‐based calculations, CCCs were calculated for these features individually. This process was repeated for all four pairs that produced four sets of CCC data per image set (individual patient). After CCC values were calculated, features with CCC >0.85 were identified. A CCC of >0.85 was chosen as the threshold to indicate a strong correlation (reproducibility).^20^, ^21^, ^22^, ^28^, ^29^, ^30^, ^31^, ^32^

In general, a CV of <10% or a CCC of >0.85 is used to indicate a radiomic feature that is reproducible across a study cohort by previous studies.^20^, ^21^, ^22^, ^24^, ^25^, ^26^, ^27^, ^28^, ^29^, ^30^, ^31^, ^32^ In this study, we applied these metrics (CV and CCC) in an opposite approach. A CV of <10% or a CCC of >0.85 indicates that a feature is “reproducible” across multiple different locations within the *same image set*. Accordingly, it is not attributed to any specific region in this image set, and in other words, it is not unique to any region.

For each image set (individual patient), the extracted features of five ROIs were compiled. The CV and CCC were then calculated for each image set and used to create a heatmap using MATLAB for later analysis.

The process defined in this study for identifying nonunique features is as follows. The first step was to flag features with CCC > 0.85. Then, if the feature was flagged in over 50% of patients for a given tissue region (adipose, heart, lung, and muscle), the feature was considered to be a failure for that tissue type. Finally, if a feature failed in two or more tissue pairs, it was considered to be nonunique as the CCC would be too high across different image sets and tissue types to provide useful information regarding the underlying tissue. This process was repeated between the image sets (patients) for both patient cohorts (GE and Siemens) to further evaluate the dependency of feature uniqueness on manufacturer and acquisition protocol.

## 3 RESULTS

We retrieved 14 patients (age 68–83 years) from GE VCT and 18 patients (age 58–68 years) from Siemens Force. The corresponding tumor size ranges from 11 to 31 mm in GE and 4 to 22 mm in Siemens. In total, 70 ROIs in GE and 90 ROIs in Siemens are used for statistical analysis. This study size is comparable to other works that investigate feature reproducibility.^20^, ^22^, ^29^

**FIGURE 2:**
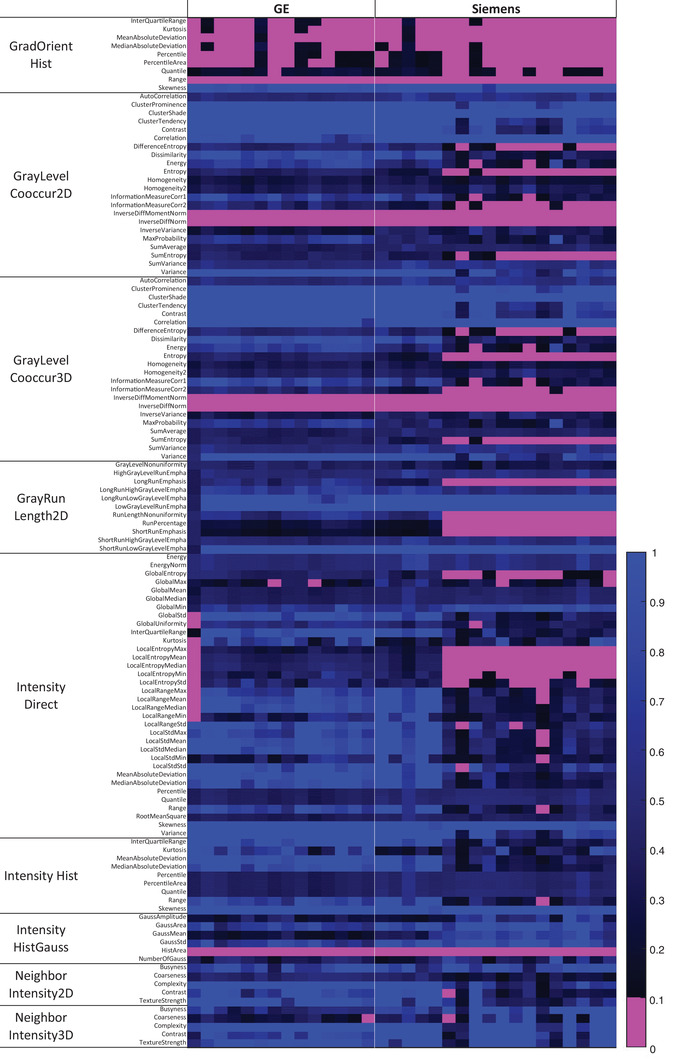
Coefficient of variation (CV) of all radiomic features among five‐tissue regions of interest (ROIs) for all image sets. Under the GE VCT, there are 14 columns representing 14 patients, whereas under the Siemens, there are 18 columns. Purple color indicates CV < 10%, and the corresponding features are considered nonunique.

Figure 2 shows an overview of the CV of radiomic features for all image sets. The CV of each feature indicates features that exhibit variation among five tissue groups. Table 1 lists the features that have CV < 10% and the times they appeared in the cohort group. Twelve features fall below the 10% CV threshold for over 50% of patients in the GE cohort image sets and 29 features in the Siemens cohort image sets. This is 9.8% and 23.6%, respectively, of the calculated radiomic features.

**TABLE 1:** List of features identified to be nonunique based on coefficient of variation (CV)

	**GE**	Patients Failed	**Siemens**	Patients Failed
Gradient Orient Histogram	InterQuartileRange	10 / 14	InterQuartileRange	16 / 18
	Kurtosis	11 / 14	Kurtosis	17 / 18
	MeanAbsoluteDeviation	13 / 14	MeanAbsoluteDeviation	17 / 18
	MedianAbsoluteDeviation	10 / 14	MedianAbsoluteDeviation	15 / 18
	Percentile	8 / 14	Percentile	13 / 18
	PercentileArea	9 / 14	PercentileArea	13 / 18
	Quantile	1 / 14	Quantile	7 / 18
	Range	14 / 14	Range	18 / 18
Gray Level Cooccurrence 2D	DifferenceEntropy		DifferenceEntropy	9 / 18
	Energy		Energy	2 / 18
	Entropy		Entropy	12 / 18
	InformationMeasureCorr1		InformationMeasureCorr1	2 / 18
	InformationMeasureCorr2		InformationMeasureCorr2	10 / 18
	InverseDiffMomentNorm	14 / 14	InverseDiffMomentNorm	18 / 18
	InverseDiffNorm	14 / 14	InverseDiffNorm	18 / 18
	SumEntropy		SumEntropy	10 / 18
Gray Level Cooccurrence 3D	DifferenceEntropy		DifferenceEntropy	9 / 18
	Energy		Energy	2 / 18
	Entropy		Entropy	13 / 18
	InformationMeasureCorr1		InformationMeasureCorr1	2 / 18
	InformationMeasureCorr2		InformationMeasureCorr2	12 / 18
	InverseDiffMomentNorm	14 / 14	InverseDiffMomentNorm	14 / 18
	InverseDiffNorm	14 / 14	InverseDiffNorm	14 / 18
	SumEntropy		SumEntropy	12 / 18
GrayRun Length2D	LongRunEmphasis		LongRunEmphasis	13 / 18
	RunLengthNonuniformity		RunLengthNonuniformity	13 / 18
	RunPercentage		RunPercentage	13 / 18
	ShortRunEmphasis		ShortRunEmphasis	13 / 18
Intensity Direct	GlobalEntropy		GlobalEntropy	9 / 18
	GlobalMax	2 / 14	GlobalMax	2 / 18
	GlobalStd	1 / 14	GlobalStd	0 / 18
	GlobalUniformity	1 / 14	GlobalUniformity	1 / 18
	Kurtosis	1 / 14	Kurtosis	
	LocalEntropyMax	1 / 14	LocalEntropyMax	
	LocalEntropyMean	1 / 14	LocalEntropyMean	13 / 18
	LocalEntropyMedian	1 / 14	LocalEntropyMedian	13 / 18
	LocalEntropyMin	1 / 14	LocalEntropyMin	13 / 18
	LocalEntropyStd	1 / 14	LocalEntropyStd	11 / 18
	LocalRangeMax	1 / 14	LocalRangeMax	6 / 18
	LocalRangeMean	1 / 14	LocalRangeMean	1 / 18
	LocalRangeMedian	1 / 14	LocalRangeMedian	1 / 18
	LocalRangeMin	1 / 14	LocalRangeMin	
	LocalRangeStd		LocalRangeStd	2 / 18
	LocalStdMax		LocalStdMax	1 / 18
	LocalStdMean		LocalStdMean	1 / 18
	LocalStdMin		LocalStdMin	1 / 18
	LocalStdStd		LocalStdStd	1 / 18
	Range		Range	1 / 18
Intensity Histogram	Range		Range	1 / 18
Intensity Hist Gauss	HistArea	14 / 14	HistArea	18 / 18
Neighbor Intensity2D	Contrast		Contrast	1 / 18
Neighbor Intensity3D	Coarseness	1 / 14	Coarseness	1 / 18

*Note*: Features that have CV < 10% for over 50% of patients are considered to be nonunique and are highlighted in red.

**FIGURE 3:**
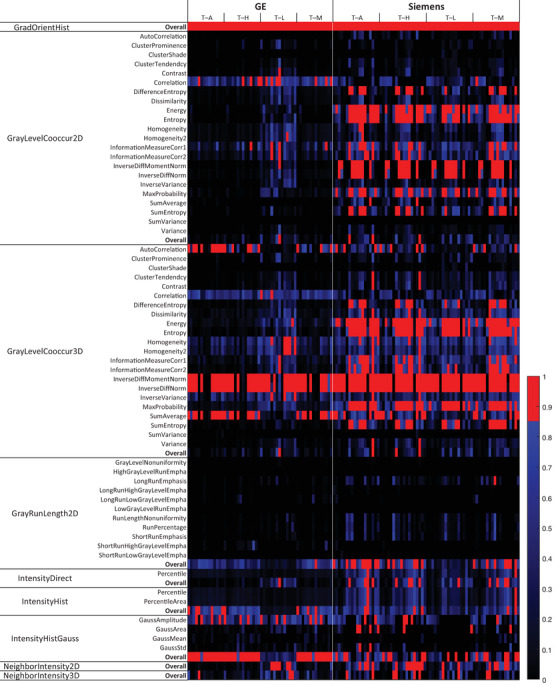
A heatmap of the concordance correlation coefficient (CCC) of radiomic features between any of four tumor/tissue pairs. Under each pair column (e.g., tumor–adipose [T–A]), 14 patients are included for GE VCT, whereas 18 patients for Siemens Force. Red color indicates CCC > 0.85, and the corresponding features are considered nonunique. T–H, tumor–heart; T–L, tumor–lung; T–M, tumor–muscle.

Figure 3 shows a heatmap of the CCC of radiomic features between the four tumor/tissue pairs (ROI_T–A_, ROI_T–H_, ROI_T–L_, and ROI_T–M_). Under each pair column (e.g., ROI_T–A_), 14 patients are included for GE VCT, whereas 18 patients for Siemens Force. Red color indicates CCC > 0.85, and the corresponding features are considered to be nonunique.

The features that are identified as nonunique based on CCC are summarized in Table 2, separated by feature group and vendor. Radiomic features that have CCC > 0.85 for over 50% of patients for a given tissue region are considered to be a failure. Any feature that fails in two or more tissue regions is considered nonunique. The nonunique features are highlighted in red. Eighteen features are identified as nonunique in the GE cohort image sets, and 16 features from the Siemens cohort image sets. This represents 14.6% and 13% of the calculated radiomic features, respectively.

**TABLE 2:** List of radiomic features identified to be nonunique based on concordance correlation coefficient (CCC)

	GE	Regions Failed	Siemens	Regions Failed
Gradient Orient Histogram	InterQuartileRange	4 / 4	InterQuartileRange	4 / 4
	Kurtosis	4 / 4	Kurtosis	4 / 4
	MeanAbsoluteDeviation	4 / 4	MeanAbsoluteDeviation	4 / 4
	MedianAbsoluteDeviation	4 / 4	MedianAbsoluteDeviation	4 / 4
	Percentile	4 / 4	Percentile	4 / 4
	PercentileArea	4 / 4	PercentileArea	4 / 4
	Quantile	4 / 4	Quantile	4 / 4
	Range	4 / 4	Range	4 / 4
	Skewness	4 / 4	Skewness	4 / 4
Gray Level Cooccurrence 2D	Energy		Energy	4 / 4
	Entropy		Entropy	3 / 4
Gray Level Cooccurrence 3D	Autocorrelation	1 / 4	Autocorrelation	
	Energy		Energy	4 / 4
	Entropy		Entropy	3 / 4
	InverseDiffMomentNorm	4 / 4	InverseDiffMomentNorm	4 / 4
	InverseDiffNorm	4 / 4	InverseDiffNorm	4 / 4
	MaxProbability		MaxProbability	4 / 4
	SumAverage	3 / 4	SumAverage	
Gray Run Length 2D	GrayLevelNonuniformity		GrayLevelNonuniformity	1 / 4
	HighGrayLevelRunEmpha		HighGrayLevelRunEmpha	1 / 4
	LongRunEmphasis		LongRunEmphasis	1 / 4
	LongRunHighGrayLevelEmpha		LongRunHighGrayLevelEmpha	1 / 4
	LongRunLowGrayLevelEmpha		LongRunLowGrayLevelEmpha	1 / 4
	LowGrayLevelRunEmpha		LowGrayLevelRunEmpha	1 / 4
	RunLengthNonuniformity		RunLengthNonuniformity	1 / 4
	RunPercentage		RunPercentage	1 / 4
	ShortRunEmphasis		ShortRunEmphasis	1 / 4
	ShortRunHighGrayLevelEmpha		ShortRunHighGrayLevelEmpha	1 / 4
	ShortRunLowGrayLevelEmpha		ShortRunLowGrayLevelEmpha	1 / 4
Intensity Hist Gauss	GaussAmplitude	3 / 4	GaussAmplitude	
	GaussArea	3 / 4	GaussArea	
	GaussMean	3 / 4	GaussMean	
	GaussStd	3 / 4	GaussStd	
	HistArea	3 / 4	HistArea	
	NumberOfGauss	3 / 4	NumberOfGauss	

*Note*: Radiomic features that have CCC > 0.85 for over 50% of patients for a given tissue region are considered to be a failure. Any feature that fails in two or more tissue regions is considered nonunique. The nonunique features are highlighted in red.

Combining CV and CCC, a total of 9 of 123 features (7.3%) are nonunique in both datasets. The common features are Inverse Difference Moment Normalized (IDMN) and Inverse Difference Normalized (IDN) from the Gray Level Cooccurrence Matrix (GLCM) 3D feature set and seven features within the Gradient Orient Histogram feature set (interquartile range, kurtosis, mean absolute deviation, median absolute deviation, percentile, percentile area, and range). For Siemens, the entropy in the GLCM 3D has CV < 10% for over 50% patients and CCC > 0.85 for three of four ROI pairs.

## 4 DISCUSSION

For the first time, this study raises the awareness of feature uniqueness in CT‐based radiomics. The assumption that radiomic features extracted from a given ROI are unique to the underlying structure may need to be reconsidered. Our test results show that more than 7% of radiomic features are not unique to a defined region for both GE and Siemens CT scanners. The hypothesis that all radiomic features extracted from an ROI are unique to the underlying structure is invalid when applied to NSCLC. Note that as we use the same ROI, it is not surprising to see histogram area in Intensity Hist Gauss (Table 1) having CV < 10%. Those features can be excluded. We also exclude shape features from the analysis for the same reason.

A GLCM describes the second‐order joint probability function of an ROI in an image. Both IDMN and IDN are calculated to measure the local homogeneity of an image. IDMN normalizes the square of the difference between neighboring intensity values by dividing over the square of the total number of discrete intensity values, whereas IDN normalizes the difference between the neighboring intensity values by dividing over the total number of discrete intensity values. The IDMN and IDN in the GLCM show consistently high CCC (>0.85) and low CV (<10%) for all image sets, independent of CT scanner. As Inverse Difference Moment (aka Homogeneity 2) and Inverse Difference (aka Homogeneity 1) are unique to the underlying structure (CCC < 0.85 and CV > 10%), the nonuniqueness of the corresponding IDMN and IDN is likely due to feature normalization, suggesting that feature calculation approaches may have an effect on feature uniqueness.

The dependence of feature uniqueness on the manufacturer can be visually observed in Figures 2 and 3. From visual inspection, more radiomic features in the Siemens scanner show high CCC (>0.85), compared to the GE scanner. The same can also be observed with the CV. It is well known that different imaging acquisition techniques heavily impact the reproducibility of radiomic features.^33^, ^34^ Our observation suggests that they may also impact radiomic feature uniqueness, which needs a further study to validate. Note that feature uniqueness is tested for each CT model separately. No radiomic features are compared across CT scanners, as generally performed by reproducibility studies.

Our study has some limitations. We extracted 123 image features using IBEX, which does not include some higher order features such as wavelet, Gabor, and Laws.^15^ Those features should be examined in the future for uniqueness. In addition, our study is limited to CT scanners from two major vendors. The observed difference in unique features suggests that the hypothesis test needs to be carried out for more CT models and vendors.

In conclusion, ∼10% of the examined radiomic features are not unique to a defined ROI for CT‐based NSCLC. Features that are not unique should be removed prior to conducting analysis, similar to removing non‐reproducible or highly correlated features. Feature uniqueness should be considered while developing radiomic models in future lung cancer studies. It is worth exploring uniqueness in other types of tumors to further expand the scope of the findings in this study.

## CONFLICT OF INTEREST

The authors declare no conflict of interest.

## AUTHOR CONTRIBUTIONS

Gary Ge and Jie Zhang conceived of the presented idea and contributed to study design. Gary Ge performed data collection, analysis, and the computations. Jie Zhang verified the analytical methods and supervised the findings of this work. All authors discussed the results and contributed to the final manuscript.

## Data Availability

The datasets used and/or analyzed in this study are available from the corresponding author upon reasonable request.
